# The role of vitamin C in the prevention of pancreatic cancer: a systematic-review

**DOI:** 10.3389/fnut.2024.1398147

**Published:** 2024-07-10

**Authors:** Samuel J. Martínez-Domínguez, Viviana Laredo, Guillermo García-Rayado

**Affiliations:** ^1^Department of Gastroenterology, Lozano Blesa University Hospital, Zaragoza, Spain; ^2^Aragón Health Research Institute (IIS Aragón), Zaragoza, Spain; ^3^School of Medicine, University of Zaragoza, Zaragoza, Spain

**Keywords:** vitamin C, ascorbate, pancreatic cancer, prevention, systematic review

## Abstract

**Introduction and aim:**

The aim of this systematic review was to assess the role of vitamin C in the prevention of pancreatic cancer (PC).

**Methods:**

A comprehensive literature search was performed in PubMed, Embase and Web of Science up to August 2023, to identify randomized controlled trials (RCT), cohort studies and mendelian randomization studies based on prospective databases assessing the role of vitamin C in PC prevention.

**Results:**

A total of twelve studies including European and North-American participants were included: two RCT, three mendelian randomization (MR) studies and seven cohort studies. Both RCT showed high quality in Cochrane risk of bias tool. Only one cohort study had <7 points in Newcastle Ottawa Scale. Both RCT found no association between the intake of 500 mg/day of vitamin C and the incidence of PC. Only one prospective cohort study found an association between vitamin C serum levels and a lower incidence of PC. The remaining cohort studies and MR studies found no association between dietary/supplements intake of vitamin C or circulating vitamin C levels and the incidence of PC.

**Conclusion:**

There is no supporting evidence that vitamin C prevents PC development. Future prospective quality studies including high-risk populations are needed.

## 1 Introduction

Pancreatic cancer (PC) is the 12th most frequent cancer and the 7th leading cause of cancer mortality (1). In addition, the incidence and mortality of PC are increasing in many developed countries, particularly among women and people 50 years or older, but also in younger patients (2). The most common histological subtype of PC is pancreatic ductal adenocarcinoma (PDAC), which represents 85% of all cases of PC (3). PDAC has one of the highest mortality rates (with a five-year survival rate around 5%) (4) due to various factors such as its difficult diagnosis (in advanced stage in most cases), its aggressive nature with strong metastatic potential and its low response rate to treatment (2).

In this context, some complementary measures such as diet and lifestyle factors have been proposed to prevent PC (3). Dietary factors, especially antioxidants like vitamin C (ascorbate) could play a role in the development of PC. Vitamin C cannot be synthesized by humans and it has to be consumed with fruits and vegetables or as a supplement (5). Vitamin C is a powerful antioxidant that inactivates free radicals-induced DNA damage. Besides, vitamin C inhibits the carcinogenic effect of nitroso compounds and can stimulate immune function (6, 7).

Despite these possible beneficial effects, the evidence of a decreased risk of PC associated with a high dietary intake of vitamin C is controversial. Some retrospective case–control studies showed an association between a high dietary vitamin C intake and a reduced risk of PC (8, 9). However, a few prospective studies have not yet confirmed this association (10, 11). Finally, two meta-analyses of observational studies showed different results. A meta-analysis by Fan et al. (12) published in 2015 found an association between a high consumption of vitamin C with a risk reduction of PC. However, another meta-analysis published by Hua et al. (13) concluded that there was not enough evidence of a relationship between the consumption of vitamin C and the prevention of PC. Noteworthy, most of the studies included in these meta-analyses were case–control studies. Case–control studies have some weaknesses as a high risk of bias, mainly recall and selection biases (14). Besides, the causal temporal relationship between exposure and outcome is not well stablished in retrospective studies.

Therefore, the aim of our study was to carry out an updated systematic review including high-quality studies to unraveal the association between the vitamin C consumption and the risk of PC.

## 2 Materials and methods

This systematic review was performed according to the Preferred Reporting Items for Systematic Reviews and Meta-Analyses 2020 (PRISMA 2020) guidelines (15). Due to the study design, institutional review board approval was not required. This systematic review was not pre-registered in any database.

### 2.1 Data sources and search strategy

A comprehensive literature search was performed in PubMed, Embase and Web of Science up to August, 2023. Database searches were performed independently by two authors using the following search strategy: vitamin C OR ascorbate. The Boolean operator “AND” was used to combine these terms with: pancreatic cancer OR cancer of pancreas OR pancreatic adenocarcinoma. No database filters were applied. All references were imported into an EndNote 20.5 reference manager file. First, EndNote automatically removed the duplicate references and, later, we manually reviewed all the references removing those corresponding to the same publication (based on title, authors and doi). Titles and abstracts were independently reviewed by all authors. Subsequently, full-text articles were reviewed again by all authors and any disparities were discussed and resolved by consensus. In addition, references cited in eligible studies were assessed to identify potential studies unnoticed in the electronic search. No additional methods were needed such as contacting authors for missing data or clarification. No language, time or other restrictions were applied.

**Figure 1:**
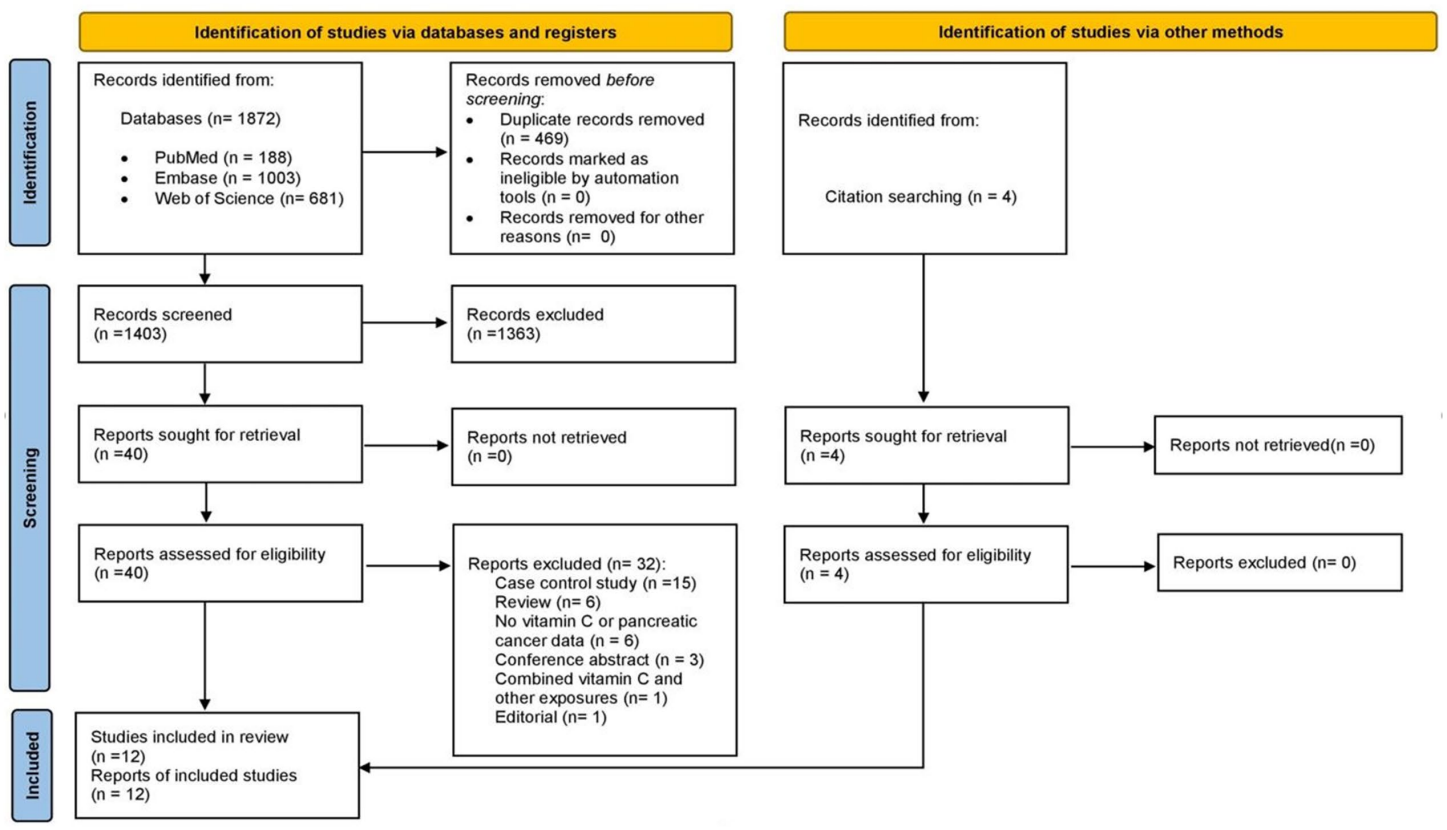
Flow chart of study selection.

A total of 1,872 records were identified from databases (PubMed 188, Web of Science 681, Embase 1,003). After an initial electronic and subsequent manual identification of duplicate records, 469 records were removed so 1,403 records were screened. During the screening by title and abstract, 1,363 references were excluded so 40 full-texts were reviewed. After applying inclusion and exclusion criteria, eight studies identified via databases were included in the systematic review (10, 16–22). The most frequent reasons for exclusion were: case control studies (*n* = 15), reviews (*n* = 6) and absence of vitamin C or PC data (*n* = 6). In addition, four reports that were not identified in electronic search were selected when citation of eligible studies were reviewed (11, 23–25). Finally, a total of twuelve studies were included in this systematic review. The flow diagram of study retrieval for this systematic review is shown in Figure 1.

### 2.2 Selection criteria

Studies were included in case of: (1) studies specifying dietary or supplement vitamin C intake or cirulating vitamin C levels; (2) studies assessing the risk of PC development during follow-up (also studies including other types of cancer if they showed PC data separately); (3) prospective observational studies, randomized controlled trials (RCT) or mendelian randomization (MR) studies based on a prospective database.

The following exclusion criteria were applied: (1) Studies reporting diet without quantifying vitamin C consumption; (2) Studies focusing on the adjuvant effect of vitamin C in the treatment of current pancreatic cancer; (3) Transversal or retrospective design; (4) Narrative reviews, systematic reviews, meta-analyses, letters, guidelines, editorials; (5) Conference communications; (6) Non-human studies; (7) *In vitro* studies.

### 2.3 Data extraction and analysis

All included studies were critically reviewed by all authors and disparities were discussed and resolved by consensus. We extracted the following variables from the included studies: publication year, study location, study design, follow-up (years), sample size (cohort, non-cancer controls and incidence of PC during follow-up), vitamin C source (dietary and/or supplements) and method of reporting this information (blood determination, written questionnaires, food diaries), age of participants, gender of participants, ethnicity, risk of PC and variables included in the adjusted analysis.

Studies whose participants reported the composition of their diet estimated the daily intake of vitamin C considering the frequency and/or amount of several foods. Later, the estimated amount of vitamin C intake was clasified by the studies into several categories. Then, the risk of incident PC during follow-up in participants with the highest amount of vitamin C intake and participants with lower amount of vitamin C intake were compared.

### 2.4 Risk of bias assessment

The Newcastle-Ottawa Scale (NOS) was used to assess the quality of cohort studies. The risk of bias assessment was initially performed by two authors and disagreements were resolved by consensus involving a third author. NOS for cohort studies is compound by 8 items (26): 4 items for the selection of exposed/non-exposed group (representativeness of the exposed cohort, selection of the non-exposed cohort, ascertainment of exposure, and outcome of interest not present at the start of the study), 1 item for the comparability of the groups and 3 items for outcome evaluation (assessment of outcome, length of follow-up period, and adequacy of follow-up). A maximum of one star can be assigned to each item except for comparability, which can be assigned two stars (maximum total score 9 stars). Studies that achieved at least 7 stars were considered to have low risk of bias.

Despite the absence of a formal tool for risk of bias assessment in MR studies (27), we used the STROBE-Mendelian Randomization checklist as a guide to address risk of bias of MR studies (28). The STROBE checklists were created to ensure clear presentation of observational studies rather than to judge the study quality of studies, and it could lead to inconsistencies in the assessment of the quality of MR studies.

The Revised Cochrane Risk of Bias Tool for randomized trials (RoB2.0) was used to assess the quality of RCT. This instrument is composed of the following five domains: Domain 1 (Risk of bias arising from the randomization process), Domain 2 (Risk of bias due to deviations from the intended interventions) (effect of assignment to intervention and effect of adhering to intervention), Domain 3 (Risk of bias due to missing outcome data), Domain 4 (Risk of bias in measurement of the outcome) and Domain 5 (Risk of bias in selection of the reported result). An overall low risk of bias was considered if the study was judged to be at low risk of bias for all domains (29).

## 3 Results

### 3.1 Study characteristics

Seven (58.3%) of the included studies were prospective cohort studies (10, 11, 16, 17, 19, 20, 25), three (25.0%) were mendelian randomization studies based on prospective cohort databases (18, 21, 22) and two (16.7%) were double-blind randomized placebo-controlled studies (23, 24). The studies were published between 1994 and 2022 and both randomized controlled studies were published in 2009 (23, 24). Six (50%) studies used data from European participants (10, 16, 18, 21, 22, 25) and six (50%) studies were conduced in North America (11, 17, 19, 20, 23, 24). Wang et al. (20) reported an observational post-trial follow-up between 2007–2011 of patients included in the Physician’s Health Study II randomized trial [Gaziano et al. (23), performed between 1997–2007]. The three MR studies used data from two European prospective databases: United Kingdom Biobank (18, 21, 22) and/or FinnGen (Finland) (18, 22). The main characteristics of the twelve included studies are shown in Supplementary Table S1.

Regarding vitamin C intake, both RCT used a daily dose of 500 mg. Only four non-RCT studies (40%) were based on circulating vitamin C or metabolites levels (16, 18, 21, 22) whereas the rest used self-reported questionnaires of diet and/or supplement vitamin C intake (60%) (10, 11, 17, 19, 20, 25).

### 3.2 Participants characteristics

Sample size of the observational studies ranged from 13,976 (19) to 309,154 participants from FinnGen (18) or 455,761 from UK Biobank (21). Regarding randomized controlled studies, Gaziano et al. (23) had a sample size of 14,641 participants (3,673 randomized to vitamin C active and vitamin E placebo, and 3,653 randomized to vitamin C and E placebo) and Lin et al. (24) had a sample size of 7,627 participants (3,824 randomized to vitamin C active and 3,803 randomized to vitamin C placebo, part of them receiving vitamin E and/or beta carotene supplements).

Seven (58.3%) studies focused on participants ≥50 years (10, 11, 17, 19, 20, 23, 25) whereas five (41.7%) studies included patients ≥40 years (16, 18, 21, 22, 24). Three studies only included males (20, 23, 25), two studies only included females (11, 24) and the three studies based on UK Biobank and FinnGen did not specify the exact proportion of patients of each gender (18, 21, 22). The rest of studies included a higher proportion of females (10, 16, 17, 19), however, male gender was more prevalent in patients with incident PC (10, 16). Five (41.7%) studies did not specified ethnicity of participants (10, 16, 20, 23, 25), whereas seven (58.3%) studies included >90% of white participants (11, 17–19, 21, 22, 24).

### 3.3 Risk of bias assessment

NOS scores for risk of bias assessment of cohort studies are shown in Supplementary Table S2 and Cochrane risk of bias tool scores for RCT are shown in Supplementary Table S3. Six studies (85.7%) had a NOS total score ≥ 7 points (10, 11, 16, 17, 20, 25) whereas only one study (14.3%) had <7 points (19). Most studies had a selection bias, mainly in the representativeness of the exposed cohort because they selected volunteers, that may not faithfully represent the community. In addition, some studies focused on postmenopausal women (11), old participants (19), male smokers (25) or male physicians (20). Only two studies (28.6%) had a star for the item “ascertainment of exposure” (16, 20), which was only awarded if the exposure was verified by an objective test or structured interview. The rest of the studies used mainly written self report.

The two RCT (23, 24) assessed with the Revised Cochrane risk of bias tool (RoB 2.0) had a low risk of bias for the five domains: risk of bias arising from the randomization process, risk of bias due to deviations from the intended interventions (effect of assignment to intervention and effect of adhering to intervention), risk of bias due to missing outcome data, risk of bias in measurement of the outcome and risk of bias in selection of the reported result.

All three MR studies (18, 21, 22) broadly followed the STROBE recommendations for methodology (study design, assumptions, statistics and assessment of assumptions) and results (description, main results and assessment of assumptions).

### 3.4 Role of vitamin C in the prevention of pancreatic cancer

Both RCT found no association between vitamin C 500 mg/day intake and the incidence of PC [Gaziano et al. (23): HR 0.97 95% CI (0.57–1.64] after 8 years of follow-up; Lin et al. (24): RR 2.32 95% CI (0.89–6.04) after 9.4 years of follow-up). In addition, Lin et al. (24) found no association between overall cancer mortality and vitamin C intake. Although both RCT used the same doses of vitamin C and placebo control, they were conducted in different populations: Gaziano et al. (23) in male physicians initially aged ≥50 years and Lin et al. (24) in women aged ≥40 years.

Banim et al. (16) was the only prospective cohort study that assessed vitamin C consumption using biomarkers (serum levels of vitamin C), and they found an association between the highest quartile of vitamin C serum levels and a lower incidence of PC [HR 0.19 95% CI (0.06–0.68)]. However, vitamin C intake was not statistically associated with PC incidence in this study [HR 0.68 95% CI (0.37–1.26)].

The remaining six prospective cohort studies and three MR studies found no association between dietary/supplements intake of vitamin C or circulating vitamin C levels and the incidence of PC (10, 11, 17–22, 25). Shibata et al. (19) had high risk of bias according to NOS so it could affect the reliability of the findings. Regarding the MR studies, although the number of included patients and the date of data extraction may be different, they probably obtained similar results because they were based on the same databases (UK Biobank and FinnGen).

Therefore, the results of each study were similar regardless of their design (RCTs, cohort studies or MR studies). The sources of vitamin C (food vs. supplements) used in the twelve studies were detailed in Supplementary Table S1, however, vitamin C sources did not have an impact on PC as neither food nor supplement intake were associated with PC. Although the median duration of follow-up ranged from 3 (20) to 16 (10) years, similar outcomes were observed. In addition, the time period used as a reference for vitamin C consumption varied between the different studies without impact on the results; for example, Wang et al. (20) periodically assessed dietary adherence but Banim et al. (16) performed a single measure of diet at baseline; and Han et al. (17), Heinen et al. (10) or Stolzenberg-Solomon et al. (25) assessed vitamin C intake over the last year. Shibata et al. (19) and Stolzenberg-Solomon et al. (25) focused on high-risk population (elderly and older males, respectively), however, they did not find association between vitamin C intake and PC. The absence of an association between vitamin C intake and PC risk is clinically significant because this finding supports not recommending a high vitamin C intake specifically for this purpose in patients with high risk of PC. However, given the pleiotropic effects of vitamin C, its intake as part of a balanced diet may be recommended in the general population for reasons other than PC.

## 4 Discussion

Given the increasing incidence and mortality of PC, the absence of adequate prevention strategies for PC, and the contradictory data on the role of vitamin C in the prevention of PC, we aimed to perform a systematic review of cohort studies, RCT and MR studies. We found no association between vitamin C intake and the risk of PC.

These results differed from those reported in the previous meta-analysis of Fan et al. (12), in which high vitamin C intake was associated with lower risk of PC, mainly because of methodological differences and new evidence (12, 21, 22). In that meta-analysis authors included thirteen case–control studies and four cohort studies, while in this systematic review we restricted to RCT, MR studies or observational studies with a prospective design (including 2 recently published) (21, 22). Moreover, other previous meta-analysis of Hua et al. (13) found a possible bias among the case–control studies that precludes drawing any conclusions; while in our review the overall risk of bias according to the NOS, Cochrane risk of bias tool and STROBE-MR was low (except for one study) (19). In addition, vitamin C intake was categorized in the pooled analysis into highest and lowest vitamin C intake groups, but vitamin C intake was graded or defined differently in each study, leading to heterogeneity.

Some studies have investigated the influence of dietary antioxidants on digestive neoplasms (30) because oxidative stress increases the risk of mutagenesis, and vitamin C intake has been proposed as a protective factor against PC, especially at high doses (3). An additional effect reducing inflammation and improving the immune function has also been suggested (16). Most evidence comes from molecular studies and case–control studies with positive results; however, when new prospective studies were developed no significant association was found (8–10, 17, 31, 32).

In the UK prospective cohort from the EPIC-Norfolk Study, the only cohort study that assessed vitamin C exposure by serum levels, there was an association between vitamin C levels and the risk of PC (16). This finding has an uncertain clinical significance since serum vitamin C levels are not determined in routine analyses. The combination of the highest quartiles of vitamin C, vitamin E and selenium decreased the risk of PC so this effect could also be mediated, at least partially, by nutrients other than vitamin C. In this study, the intake of vitamin C was determined using dietary questionnaires and blood samples at baseline. There was no association between PC and low vitamin C intake based on questionnaires at 10 years and at 17 years follow-up. However, the quartile with the highest serum concentration of vitamin C was associated with a lower risk of PC at 10 years but not at 17 years. It should be noted that diet data and blood samples were collected baseline, but dietary patterns and nutrient’s concentrations can change over time. Despite results were adjusted by smoking status, body mass index, age and diabetes, there may be other confounding factors which can influence the risk (genetic variants, chronic pancreatitis, family history of PC.) (33). Moreover, some conditions such as alcohol consumption (which can lead to chronic pancreatitis) may influence the dietary pattern leading to vitamin C deficiency (34). Despite its large sample size and accurate measurement of exposure and outcome, the clinical significance of a lower risk of PC in patients with higher serum vitamin C levels remains unclear because no association with daily intake was found.

Contrary to the UK cohort, in the US VITAL cohort study, including 184 cases of pancreatic adenocarcinoma during the follow-up, there was no association between vitamin C consumption and the risk of PC (17). In this study, data from vitamin C was obtained from questionnaires and results were adjusted by the main risk factors such as diabetes, age, smoking status, alcohol consumption and family history of PC. In this cohort, smokers had a significantly lower vitamin C consumption comparing with non-smokers (mean 104.5 mg vs. 125.5 mg, *p* < 0.0001). In a Dutch cohort including 423 cases of PC, no association was found between vitamin C intake based on questionnaires and PC after adjustment for main confounding factors such as family history of PC, smoking status and alcohol consumption (10). Another study with the Iowa Women’s Health cohort (256 PC) and a similar design, also failed in finding association between vitamin C an PC (11). Therefore, there is cumulative evidence from prospective cohort studies carried out in different populations that supports that there is no association between vitamin C intake and the risk of PC.

The most recently published prospective studies assessing this issue are MR studies, which are designed to minimize the risk of bias due to a randomized assign of genetic variants. Moreover, Zhang et al. (22) included 2 different populations in their study (UK and Finnish database) and performed a meta-analysis with consistent results. Two instrumental variables were also included (metabolites and antioxidant levels). In this study, despite the possible association between vitamin C metabolites and PC in the UK sample, those results were not found in neither of the meta-analysis for metabolites and for antioxidant levels. In the MR study of Yin et al. (21), including also data from the UK biobank, no association was found between vitamin C (metabolites and antioxidant) and the risk of PC using the inverse-variance weighted method and performing meta-analysis. In this study there was not significantly horizontal pleiotropy, which minimizes the risk of bias. However, authors admitted that the design of the study is unable to determine if some subpopulations of high risk (for example smokers or family history of PC) would benefit from vitamin C supplementation.

The main risk factors for PC are age, sex, family history, type-2 diabetes mellitus, obesity, smoking habit, risky alcohol consumption and chronic pancreatitis (35). In fact, some studies suggest that the benefit of vitamin C could be limited to high risk populations (36). However, Stolzenberg-Solomon et al. (25) published results from a male smokers Finnish cohort with no differences in PC according vitamin C intake (median vitamin C daily intake in PC 82.8 mg vs. 87 mg in non-PC, *p* = 0.20, 25). Besides, in a US cohort of elderly patients published by Shibata et al. (19), higher vitamin C intake was not associated with a statistically significant reduced risk of PC.

Given the late diagnosis of PC, the presence of advanced disease such as bone metastases at diagnosis is not uncommon, and they can also appear during follow-up. The combination of nitrogen-containing bisphosphonates such as zoledronic acid with nab-paclitaxel reduced peritoneal dissemination, fibrosis, cell proliferation and angiogenesis in a preclinical study (37). Additionally, zolendronic acid showed antitumor activity toward pancreatic ductal adenocarcinoma and stimulated the antitumor response of immune system by activating of γδ-type T cell receptors, influencing the median time to the first skeletal-related events (38, 39). In addition, the efficacy of zolendronic acid also appears to involve innate immunity, particularly through tumor-associated macrophages (TAM) (38).

To our knowledge this is the most up-to-date systematic review addressing this topic. Although the exclusion of transversal studies might have left out some information, comparing with case–control studies the prospective cohorts analyzed include a high number of cases and its design minimizes the risk of recall and selection bias. When asking about vitamin C intake in patients with PC, there is a risk of overestimation of the association and, moreover, changes in eating behavior secondary to symptoms are expected. In addition, the studies included in our systematic review reported similar outcomes for both food and supplement sources of vitamin C and the results were adjusted by relevant confounding factors such as age, sex, alcohol consumption or smoking habit. Due to the rapid mortality in this type of cancer, case–control studies are also at risk of selection bias. However, cohort studies included in this review have also some limitations. Firstly, most of them get information about vitamin C intake using questionnaires, which are at risk of bias and are less accurate than blood test, since other factors may influence in the bioavailability of the antioxidant. However, this risk of bias extends to the entire cohort and is not only limited to cases. Moreover, the highest quality studies included in this review are based on vitamin C serum levels and its design could be comparable to RCT. Secondly, most of the studies included assessed vitamin C intake at baseline and not in the follow-up, but dietary patterns may change throughout life. Thirdly, only two studies are based on high risk cohorts (elderly and smokers), so it is difficult to extend conclusions to that populations. Fourth, the mean follow-up of the RCT or prospective cohort studies may be insufficient to accurately detect incident PC, and the results of the studies are not adjusted for the same variables. Fifth, the systematic review was not pre-registered in any database. Sixth, a wide range of study designs were included leading to potential heterogeneity that could influence interpretation of results. Finally, all cohorts included in the review are from Europe or USA, so these results cannot be generalized to patients from other geographic areas.

Our findings support that vitamin C consumption cannot be generally recommended for PC prevention. Vitamin C consumption should be included as part of a balanced diet for other reasons but increasing its consumption would not be justified for an eventual reduction in the risk of PC. Future prospective and long-term studies are needed to elucidate the effect of vitamin C in the prevention of PC in high-risk populations such as patients with history of chronic pancreatitis, smokers or family history of PC.

In conclusion, in this systematic review which includes the most recent and highest-quality methodological studies published to date, no association was found between vitamin C and PC risk. However, more prospective quality studies including high-risk populations are needed to assess this issue.

## Data availability statement

The original contributions presented in the study are included in the article/Supplementary material, further inquiries can be directed to the corresponding author.

## Author contributions

SM-D: Conceptualization, Data curation, Methodology, Writing – original draft. VL: Conceptualization, Data curation, Methodology, Writing – original draft. GG-R: Conceptualization, Data curation, Methodology, Supervision, Writing – review & editing.
