# Supplementary material for: The role of vitamin C in the prevention of pancreatic cancer: a systematic-review
Source: Front Nutr. 2024 Jul 10;11:1398147. doi: 10.3389/fnut.2024.1398147 (PMC11285103; doi:10.3389/fnut.2024.1398147)
Supplement: Supplementary file 1 [file Table_1.DOCX]

Supplementary Material

***Table 1***. Main characteristics of the twelve studies included in the systematic review.

| **Study reference** | **Location** | **Design** | **Follow-up duration** | **Total sample size** | **Cases of PC** | **Vitamin C assessment** | **Age** (years) | **Gender**  (male)  n (%) | **Ethnicity** | **Outcome** (risk of PC) | **Adjusted variables** |
| --- | --- | --- | --- | --- | --- | --- | --- | --- | --- | --- | --- |
| **Banim, 2013** | UK | Prospective Cohort Study  Participants recruited into the European Prospective Investigation of Cancer-Norfolk Study (EPIC-Norfolk) | 10-17 years  Recruited between 1993-1997 | 3,970 non-cancer controls  Cohort: 23,658 | 49 incident PC during first 10 years of follow-up (86 during first 17 years) | Vitamin C measurement in serum samples and 7-day food diaries | 40-74 years  PC: 64.2±8.3  Controls: 59.3±9.4 | Overall cohort: 55% female  PC: 27 (55.1%)  Controls: 1,740 (43.8%) | NS | Highest quartile of vitamin C serum levels: HR 0.19 95%CI (0.06-0.68)  Sumation of three higher quartiles of vitamin C intake in diaries: HR 0.68 95CI% (0.37-1.26) | Age at recruitment, gender, smoking, DM, BMI, total energy intake and use of supplements containing vitamin C |
| **Gaziano, 2009** | USA | Randomized, double-blind, placebo-controlled trial | 8 years  1997-2007 | 3,673 randomized to Vitamin C active and Vitamin E placebo  3,653 randomized to vitamin C and E placebo  Cohort: 14,641 male physicians initially aged ≥ 50 years | 27 PC active Vitamin C  28 PC placebo | Intervention: Vitamin C 500 mg/day | Overall cohort: 64.3±9.2 | Only males | NS | HR= 0.97 95% CI (0.57-1.64), p= 0.91 | Age, cohort or the study (I or II) |
| **Han, 2013** | USA | Prospective Cohort Study  Participants recruited into the VITamins And Lifestyle (VITAL) cohort | Median 7.1 years  Recruited between 2000-2002 | Cohort: 77,446 | 184 incident PC | Written questionnaire of diet and 10-year supplement intake | 50-76 years  Overall cohort 62.0±7.5 | Overall cohort:  37,221 (48.1%) | Overall cohort:  White 72,220 (93.3%)  Others 5,226 (6.7%) | Dietary vitamin C intake:  aHR 0.89 95%CI (0.58-1.35), p=0.51  Vitamin C supplement intake:  aHR 0.82 95%CI (0.56-1.19), p=0.44  Total intake (diet + supplements): aHR 0.82 95%CI (0.56-1.21), p=0.46 | Age, gender, ethnicity, education, total energy intake, BMI, physical activity, smoking status, alcohol consumption, family history of PC and medication for DM |
| **Heinen, 2012** | The Netherlands | Prospective Cohort Study  Participants recruited into The Netherlands Cohort Study (NLCS) | 16.3 years  Recruited in 1986 | Subcohort: 3,868  Cohort: 120,852 | 423 incident PC | Semi-quantitative questionnaire of food consumption during the previous year and 5-year supplement intake | 55-69 years  PC: 62.1±4.1  Subcohort: 61.3±4.2 | PC: 223 (52.7%)  Subcohort: 1,884 (48.7%) | NS | Dietary vitamin C intake: aHR 1.00 95%CI (0.98-1.03), p=0.74  Vitamin C supplement intake:  aHR 0.83 95%CI (0.58-1.18) | Age, gender, smoking, BMI, intake of energy, coffee, alcohol, red meat, family history of PC and DM |
| **Inoue-Choi, 2011** | USA | Prospective Cohort Study  Postmenopausal women of the Iowa Women´s Health Study (IWHS) | The study began in 1986. Until the end of 2007 | Cohort: 34,642 | 256 incident PC | Self-administered questionnaire of food consumption | 55-69 years  PC: 74 (28.9%) <60 years, 91 (35.6%) 60-65 years, 91 (35.6%) ≥65 years | Only females | White 250 (99.6%) | Highest vitamin C intake:  aHR 0.99 95%CI (0.66-1.49)  p trend=0.84 | Age, race, education, alcohol, smoking, physical activity, BMI and DM |
| **Larsson, 2022** | Finland and UK | Mendelian randomization study  Data from FinnGenn and UK Biobank | UK biobank: since recruitment 2006-2012  FinnGen: started in 2017, data until freeze 7 | FinnGen: 309,154  UK Biobank: 367,542 | - | Genetically predicted circulating vitamin C levels | UK biobank: 40-69 years at recruitment  Finngen: median age 63 years | NS | UK biobank: 20,000 of 500,000 non-European ancestries (living in UK) | Pooled data of the UK biobank and FinnGen:  OR 1.04 95% CI (0.72-1.49) | Meta-analysis combined estimates for both databases |
| **Lin, 2009** | USA | Double-blind, placebo-controlled trial  Women´s Antioxidant Cardiovascular Study | 9.4 years  Randomized between 1995 and 1996 | Cohort: 7,627 women: 3,824 vitamin C active (+ vitamin E 2,041 and + beta carotene 2,043) and 3,803 vitamin C placebo (+ vitamin E 2,042 and + beta carotene 2,041) | Incident PC Vitamin C active: 14  Incident PC Vitamin C placebo: 6 | Intervention:  Vitamin C 500 mg/day  Questionnaires. PC diagnosis and deaths were confirmed by medical record review | ≥ 40 years  Vitamin C active: 60.4±9  Vitamin C placebo: 60.4±9 | Only females | Caucasian:  Vitamin C active 94.0%/ placebo 93.9%  African American:  Vitamin C active 3.3%/placebo 3.3% | PC incidence: RR 2.32 95% CI (0.89-6.04)  Cancer mortality: RR 1.28 95% CI (0.95-1.73) | Age, main effect terms of the 3 antioxidants (vitamin C, vitamin E and beta carotene), smoking status, alcohol consumption, BMI |
| **Shibata, 1994** | USA | Prospective Cohort Study  Elderly | 9 years  The study began in 1981. Until June 1990 | Cohort: 13,976 members contributed 100,921 persons-years of follow-up | 65 incident PC (28 males and 37 females) | Self-reported diet and use of vitamin supplements | 80% of the cohort between 65-85 years  Males: 75.0±7.2  Females: 73.8±7.4 | Females: 66,826 person-years  Males: 34,095 person-years | White almost all | Highest dietary vitamin C intake:  aRR 0.79 95%CI (0.44-1.43) | Age, gender and smoking |
| **Stolzenberg-Solomon, 2002** | Finland | Prospective Cohort Study  Older male smokers of the Alpha-Tocopherol, Beta-Carotene Cancer Prevention Study (ATBC Study) | Median 10.2 years.  Recruitment 1985-1988.  Until 1997 | 26,948 non-cancer controls  Cohort: 27,111 | 163 incident PC | Self-administered dietary questionnaire | 50-69 years  *PC: 58 (55-62)  *Non-cancer controls: 57 (53-61) | Only males | NS | Vitamin C intake (mg/day):  *PC: 82.8 (63.0-112.5)  *Non-cancer controls: 87 (66-115)  p=0.20 | Age, smoking, education, occupational physical activity, dietary folate, saturated fat and carbohydrate intakes, history of DM. |
| **Wang 2014** | USA | Observational follow-up between 2007-2011  Post-trial follow-up in the Physician´s Health Study II randomized trial[27] | 2.8 ± 3.8 years | Vitamin C active: 7,329  Vitamin C placebo: 7,312  Cohort: 14,641 male physicians initially aged ≥ 50 years | 9 PC Vitamin C active  13 PC Vitamin C placebo | Questionnaires every 6 months during the first year and annually thereafter.  Medical records review (PC diagnosis and mortality) | Vitamin C active: 64.3 ±9.2  Vitamin C placebo: 64.3 ± 9.1 | Only males | NS | PC: HR 0.70 95% CI (0.30-1.64)  PC deaths: HR 0.50 95% CI (0.22-1.18) | Age, cohort of the study and other randomized assignments |
| **Yin, 2022** | UK | Mendelian randomized study  Data from UK Biobank prospective database | Since recruitment 2006-2012 | 455,761 non-cancer controls | 587 PC | Diet-derived absolute circulating antioxidants and their metabolites | 40-69 years at recruitment | NS | 20,000 of 500,000 non-European ancestries (living in UK) | For absolute circulating antioxidants:  OR 0.99 95%CI (0.94-1.04), p=0.74  For circulating antioxidants metabolites:  OR 1.22 95%CI (0.85-1.75), p=0.28 | NS |
| **Zhang, 2022** | UK and Finland | Two-sample mendelian randomization study  Data from UK Biobank and FinnGen | UK biobank: since recruitment 2006-2012  FinnGen: started in 2017 | UK biobank:  419,598 non-cancer controls  FinnGen study:  174,006 non-cancer controls | UK biobank:  933 PC  FinnGen study:  605 PC | Diet-derived absolute circulating antioxidants and their metabolites | UK biobank: 40-69 years at recruitment  Finngen: median age 63 years | NS | UK biobank: 20,000 of 500,000 non-European ancestries (living in UK) | Pooled data of the UK biobank and FinnGen:  OR 1.07 95%CI (0.73-1.57), p=0.741 | Meta-analysis combined estimates for both databases |

BMI: Body Mass Index. DM: Diabetes Mellitus. NS: Not Specified. PC: Pancreatic Cancer. UK: United Kingdom. USA: United States of America. *Median and interquartile range.

***Table 2***. Risk of bias assessment of the seven cohort studies included in the systematic review according to the Newcastle-Ottawa Scale (NOS).

| **Study reference** | **SELECTION** | | | | **COMPARABILITY** | **OUTCOME** | | | **NOS total score (maximum 9 points)** |
| --- | --- | --- | --- | --- | --- | --- | --- | --- | --- |
|  | **Representativeness of the exposed cohort** | **Selection of the non-exposed cohort** | **Ascertainment of exposure** | **Outcome of interest not present at start of the study** |  | **Assessment of outcome** | **Length of follow-up period** | **Adequacy of follow-up** |  |
| **Banim, 2013** |  | 🟌 | 🟌 | 🟌 | 🟌🟌 | 🟌 | 🟌 | 🟌 | **8** |
| **Han, 2013** |  | 🟌 |  | 🟌 | 🟌🟌 | 🟌 | 🟌 | 🟌 | **7** |
| **Heinen, 2012** |  | 🟌 |  | 🟌 | 🟌🟌 | 🟌 | 🟌 | 🟌 | **7** |
| **Inoue-Choi, 2011** |  | 🟌 |  | 🟌 | 🟌🟌 | 🟌 | 🟌 | 🟌 | **7** |
| **Shibata, 1994** |  | 🟌 |  |  | 🟌 | 🟌 | 🟌 | 🟌 | **5** |
| **Stolzenberg-Solomon, 2002** |  | 🟌 |  | 🟌 | 🟌🟌 | 🟌 | 🟌 | 🟌 | **7** |
| **Wang, 2014** |  | 🟌 | 🟌 | 🟌 | 🟌🟌 | 🟌 | 🟌 | 🟌 | **8** |

NOS[16]: Newcastle-Ottawa Scale. Single star: 1 point. Double stars: 2 points. A maximum of one star can be assigned to each item except for comparability, which can be assigned two stars (maximum total score 9 stars).

***Table 3***. Risk of bias assessment of the two randomized controlled trials included in the systematic review according to the Revised Cochrane Risk of Bias Tool for randomized trials (RoB 2.0).

| **Domain/signalling questions** | **Gaziano, 2009** | **Lin, 2009** |
| --- | --- | --- |
| **Domain 1: Risk of bias arising from the randomization process** | | |
| 1.1 Was the allocation sequence random? | Y | Y |
| 1.2 Was the allocation sequence concealed until participants were enrolled and assigned to interventions? | Y | Y |
| 1.3 Did baseline differences between intervention groups suggest a problem with the randomization process? | N | N |
| **Risk of bias judgement** | **Low risk** | **Low risk** |
| **Domain 2: Risk of bias due to deviations from the intended interventions (effect of assignment to intervention)** | | |
| 2.1 Were participants aware of their assigned intervention during the trial? | N | N |
| 2.2 Were carers and people delivering the interventions aware of participant´s assigned intervention during the trial? | N | N |
| 2.3 If Y/PY/NI to 2.1 or 2.2: Were there deviations from the intended intervention that arose because of the trial context? | - | - |
| 2.4 If Y/PY to 2.3: Were these deviations likely to have affected the outcome? | - | - |
| 2.5 If Y/PY/NI to 2.4: Were these deviations from intended intervention balanced between groups? | - | - |
| 2.6 Was an appropriate analysis used to estimate the effect of assignment to intervention? | Y | PY |
| 2.7 If N/PN/NI to 2.6: Was there potential for a substantial impact (on the result) of the failure to analyse participants in the group to which they were randomized? | - | - |
| **Risk of bias judgement** | **Low risk** | **Low risk** |
| **Domain 2: Risk of bias due to deviations from the intended interventions (effect of adhering to intervention)** | | |
| 2.1 Were participants aware of their assigned intervention during the trial? | N | N |
| 2.2 Were carers and people delivering the interventions aware of participant´s assigned intervention during the trial? | N | N |
| 2.3 [If applicable] If Y/PY/NI to 2.1 or 2.2: Were important non-protocol interventions balanced across intervention groups? | - | - |
| 2.4 [If applicable] Were there failures in implementing the intervention that could have affected the outcome? | PN | PN |
| 2.5 [If applicable] Was there non-adherence to the assigned intervention regimen that could have affected participants outcomes? | N | N |
| 2.6 If N/PN/NI to 2.3, or Y/PY/NI to 2.4 or 2.5: Was an appropriate analysis used to estimate the effect of assignment to intervention? | - | - |
| **Risk of bias judgement** | **Low risk** | **Low risk** |
| **Domain 3: Risk of bias due to missing outcome data** | | |
| 3.1 Were data for this outcome available for all, or nearly all, participants randomized? | Y | PY |
| 3.2 If N/PN/NI to 3.1: Is there evidence that the result was not biased by missing outcome data? | - | - |
| 3.3 If N/PN to 3.2: Could missingness in the outcome depend on its true value? | - | - |
| 3.4 If Y/PY/NI to 3.3: Is it likely that missingness in the outcome depended on its true value? | - | - |
| **Ris of bias judgement** | **Low risk** | **Low risk** |
| **Domain 4: Risk of bias in measurement of the outcome** | | |
| 4.1 Was the method of measuring the outcome inappropriate? | N | N |
| 4.2 Could measurement or ascertainment of the outcome have differed between intervention groups? | N | N |
| 4.3 If N/PN/NI to 4.1 and 4.2: Were outcome assessors aware of the intervention received by study participants? | N | N |
| 4.4 If Y/PY/NI to 4.3: Could assessment of the outcome have been influenced by knowledge of intervention received? | - | - |
| 4.5 If Y/PY/NI to 4.4: Is it likely that assessment of the outcome was influenced by knowledge of intervention received? | - | - |
| **Risk of bias judgement** | **Low risk** | **Low risk** |
| **Domain 5: Risk of bias in selection of the reported result** | | |
| 5.1 Were the data that produced this result analysed in accordance with a pre-specified analysis plan that was finalized before unblinded outcome data were available for analysis? | Y | Y |
| Is the numerical result being assessed likely to have been selected, on the basis of the results, from... | | |
| 5.2 ... multiple eligible outcome measurements within the outcome domain? | PN | PN |
| 5.3 ... multiple eligible analyses of the data? | PN | PN |
| **Risk of bias judgement** | **Low risk** | **Low risk** |
| **Overall risk of bias judgement** | **Low risk** | **Low risk** |
